# Different Influences of Bacterial Communities on Fe (III) Reduction and Phosphorus Availability in Sediments of the Cyanobacteria- and Macrophyte-Dominated Zones

**DOI:** 10.3389/fmicb.2018.02636

**Published:** 2018-11-14

**Authors:** Xianfang Fan, Shiming Ding, Mengdan Gong, Musong Chen, ShuaiShuai Gao, Zengfeng Jin, Daniel C. W. Tsang

**Affiliations:** ^1^State Key Laboratory of Lake Science and Environment, Nanjing Institute of Geography and Limnology, Chinese Academy of Sciences, Nanjing, China; ^2^Graduate School of the Chinese Academy of Sciences, Beijing, China; ^3^Department of Civil and Environmental Engineering, Faculty of Construction and Environment, The Hong Kong Polytechnic University, Kowloon, China

**Keywords:** bacterial abundance and community composition, dissolved reactive phosphorus, iron reduction, freshwater sediment, HR-Peeper, eutrophication

## Abstract

Little is known about the effects of bacterial community on iron (Fe) and phosphorus (P) cycles in sediments under different primary producer habitats in different seasons. Lake Taihu has both the cyanobacteria- and macrophyte-dominated lake zones. In this work, the abundance and structure of bacterial community was investigated using qPCR and 16S rRNA gene high throughput sequencing, respectively. Compared with the sediments in the cyanobacteria-dominated lake zone, sediments in the macrophyte-dominated lake zone had higher TP, TOC and TN contents but lower DO and Eh values. Dissolved reactive P, dissolved Fe, and their molar ratios (Fe/P) were lower in the sediments of the cyanobacteria-dominated lake zone than those in the macrophyte-dominated lake zone. Consistent with this was the significantly lower abundance of total and typical Fe redox transforming bacteria in the sediment of the cyanobacteria-dominated lake zone than those in the macrophyte-dominated lake zone. Correlation analyses also revealed positive influence of abundances of total bacteria and typical Fe reducing bacteria on dissolved Fe and Fe/P ratio. The results showed that, in the cyanobacteria-dominated open water zone, *Acidimicrobiaceae* was capable of Fe metabolism, contributing to higher P flux in summer. In the cyanobacteria-dominated bay, Sva0081 sediment group and *Desulfobulbaceae* could transform sulfate to sulfide, which resulted in the reduction of Fe (III), while in the macrophyte-dominated zones, *Clostridium* sensu stricto 1 could couple oxidation of organic carbon with the reduction of Fe (III). The present study adds new knowledge linking the bacterial communities with the physicochemical cycles of Fe and P in sediments under different primary producer habitats.

## Introduction

In freshwater systems, phosphorus (P) is a key limiting nutrient (Schindler, 2006), and excessive P loading often results in eutrophication and cyanobacterial blooms. Reducing the external P loading has not always resulted in a rapid recovery from eutrophication (Qin et al., 2015; Yang et al., 2016). Such delayed responses were typically caused by internal P release from the sediment (Sondergaard et al., 2013). Reactive iron (Fe) was identified as a key factor in regulating the mobility of P in sediments (Ding et al., 2016). Decomposition of biomass from primary production, such as cyanobacterial biomass, could fuel anoxia in sediments. Under anaerobic conditions, Fe (III) could be reduced directly by microbes or by sulfide, which was mainly produced through sulfate reduction (Rozan et al., 2002). With the reduction of Fe (III) to Fe (II), P that was previously bound to Fe (III) phases, was released from the sediments (Dellwig et al., 2010). Despite important implications of Fe and P cycling in lakes, a detailed assessment of seasonal changes in Fe and P under different lake trophic statuses with different primary producers is still lacking.

Microorganisms play a key role in many reactions of biogeochemical cycles, involving Fe and P in sediments. A broad diversity of microorganisms catalyzes redox transformations of Fe (Weber et al., 2006). For example, under neutral pH, Fe (II)-oxidizing bacteria (FOB) belonging to *Gallionellaceae* family (genera *Gallionella* and *Sideroxydans*), *Burkholderiales*-related genus *Leptothrix*, *Rhodocyclaceae*, and *Comamonadaceae* (genus *Comamonas*) were detected in freshwater sediments (Emerson et al., 2010). In comparison, Fe (III)-reducing bacteria (FRB) couple oxidation of organic carbon or H_2_ gas with the reduction of dissolved and solid-phase Fe (III) species (Konhauser et al., 2011). They belong to a diverse group that typically includes genera, such as *Geobacter, Shewanella, Geothrix*, and *Crenothrix* in freshwater sediments. Higher abundance of sulfate reducing bacteria was simultaneously observed with the high release of dissolved Fe and reactive P in the surface sediment of the cyanobacteria-dominated zones (Chen et al., 2016). Under circumneutral pH conditions, Fe mobility relies on its oxidation state, and therefore, Fe redox transformations mediated by bacteria can cause precipitation or dissolution of Fe-minerals.

Lake Taihu provides an ideal backdrop to investigate the potential interactions between bacterial community and Fe and P cycles in sediments. Lake Taihu is a shallow and large freshwater lake in China (with the mean depth of 1.9 m and the area of 2338 km^2^), and has a watershed that provides home to 30 million residents (Qin et al., 2010). Anthropogenic disturbances have influenced the lake for several decades and the integrity of its ecosystem has significantly changed. This lake can be subdivided into the cyanobacteria- and macrophytes-dominated lake zones. The open waters and bays in the north of Lake Taihu were dominated by cyanobacteria (for example, the Meiliang Bay) (Luo et al., 2014). Cyanobacterial blooms are especially severe in summer seasons, and often occur in these areas. Due to long-term riverine discharges of municipal/industrial wastewater that drain into the northern bays, these areas have higher internal P loading than the open waters, and therefore, suffer from severer cyanobacterial blooms (Ding et al., 2015). On the other hand, the lake zones dominated by macrophyte are mainly located at the eastern bays, and include the East Taihu Bay, Xukou Bay, and Gonghu Bay (Luo et al., 2014). Previous studies indicate significant heterogeneity of microbial community and Fe reduction rate between the sediments of two zones (Fan and Wu, 2014; Chen et al., 2015; Chen and Jiang, 2016; Fan and Xing, 2016). However, the effects of bacterial community on Fe and P cycles in the two habitat types of Lake Taihu remain poorly understood.

In this study, quarterly sediment sampling was carried out at three sites in Lake Taihu. The high-resolution dialysis (HR-Peeper) technique was used to determine dissolved reactive P (SRP) and dissolved Fe profiles at a vertical resolution of 4.0 mm. The abundance and composition of bacterial community were investigated using qPCR and 16S rRNA high throughput sequencing, respectively. The aim was to investigate how bacterial community affects the Fe and P cycling in the sediment under different habitats and seasons.

## Materials and Methods

### Site Description and Sample Collection

**FIGURE 1:**
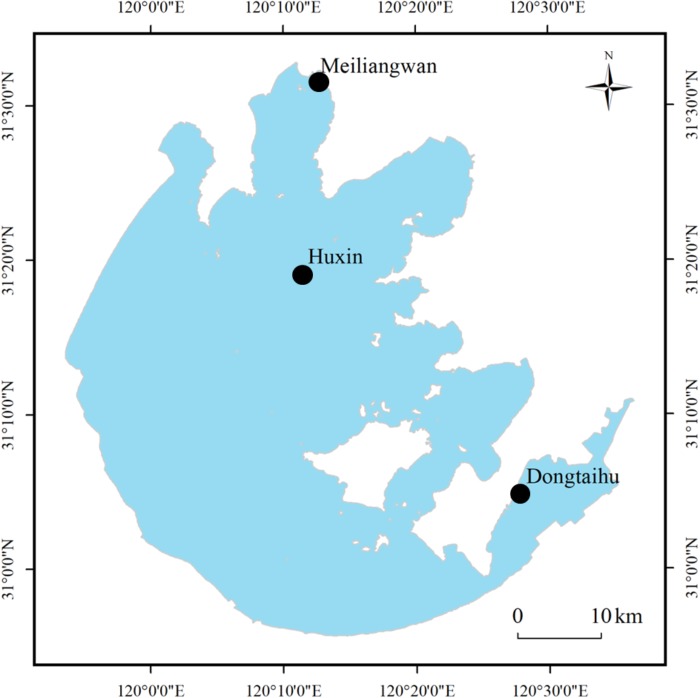
Location of sampling sites in Lake Taihu. Site Meiliangwan represent the cyanobacteria-dominated bay. Huxin belongs to cyanobacteria-dominated open water and site Dongtaihu is associated with a macrophyte-dominated bay.

In Lake Taihu, three sampling sites were selected (see Figure 1), which belonged to three different ecological types. The first sampling site, Huxin (31°19.225’ N, 120°11.563’ E), was in the open water zone and was less disturbed by the cyanobacterial blooms. The second sampling site, Meiliangwan (31°31.558’N, 120°12.567’E), was in Meiliang Bay and was seriously affected by cyanobacterial blooms. The third site, Dongtaihu (31°4.977’ N, 120°27.969’E), was dominated by the aquatic macrophyte (Luo et al., 2014).

Nine replicate sediment cores were collected from the sampling sites (Huxin, Meiliangwan, Dongtaihu) on April 29, July 18, October 17 of 2016 and January 10 of 2017. A gravity corer (11 cm × 50 cm, EasySensor Co., China) was used to collect the sediment cores. Within 4 h, the sediment cores were transported back to the laboratory. Three cores were placed in a tank (47 cm × 47 cm × 40 cm), which was maintained at 20°C in April, at 29.5°C in July, at 18.8°C in October and at 8.1°C in January. These temperatures were based upon the lake water temperature in respective months, and were used to employ HR-Peeper probes. Another set of three cores was used to determine the vertical distributions of DO and Eh on the same sampling day. Furthermore, the three cores were sliced and used for sediment bacterial community and physiochemical analysis. Specifically, the upper 0–100 mm of the three sediment cores were sliced in 10 mm increments and thoroughly homogenized. Previous studies have shown that the sediment interval between −20 and −30 mm depth was important Fe reduction zones, where abundant sediment SRP was released, especially during the summer seasons (Ding et al., 2015, 2016; Chen et al., 2016). Therefore, in this study, these sediment sections (−20 to −30 mm) were used for DNA extraction and analysis of chemical properties (see proceeding sections). The samples used to analyze the bacterial communities were stored at −50°C, while those used to analyze the physiochemical parameters were stored at −20°C.

### Preparation and Deployment of the HR-Peeper

The HR-Peeper was used to measure SRP and dissolved Fe profiles at a vertical resolution of 4.0 mm. This device consists of 30 equally spaced 200 μL chambers. Prior to deployment, each chamber of the probe was filled with deionized water, and the surface of the chambers was covered with a 0.45 μm cellulose nitrate membrane. The assembled HR-Peeper was deoxygenated using N_2_ flushing overnight. Then, the probe was deployed in the sediments for 48 h to obtain equilibrium between the concentrations of dissolved substances in the pore and the chamber water. After retrieval, about 200 μL pore water sample was immediately collected from each chamber for analyzing SRP and dissolved Fe (Chen et al., 2016).

### Physiochemical Analysis

The Multi-Parameter Water Quality Sonde was used to investigate the physiochemical parameters of overlying water (YSI 6600, United States; Supplementary Table S1). The concentrations of SRP and dissolved Fe in HR-Peeper were detected using molybdenum blue (Murphy and Riley, 1962) and phenanthroline colorimetric method (Tamura et al., 1974), respectively. The concentrations of DO and Eh were measured using oxygen and redox microelectrodes (OX-100 and RD-100, Unisense, Denmark). The sediment total phosphorus (TP), total nitrogen (TN), total organic carbon (TOC), and loss on ignition (LOI) were analyzed following the methods described previously (Fan and Xing, 2016).

### DNA Extraction and qPCR

The sediment samples were freeze-dried at −53°C using a freeze-dryer (Labconco FreeZone Triad 2.5 L, United States). Then, the DNA was extracted from 0.5 g of freeze-dried sediments using FastDNA spin kit (MP Biomedicals LLC, OH, United States). Between 2 and 5 ng of extracted DNA was subjected to SYBR Green-based quantitative PCR inside a CFX96 Optical Real-Time Detection System (Bio-Rad Laboratories, Inc., Hercules, United States) using primers Eub341F and Eub515R (Simmons et al., 2007). The qPCR assay was carried out in 20 μL reaction volume containing 10 μL of SYBR^®^ Premix Ex Taq (TaKaRa Biotech, Dalian, China), 0.25 μM of each primer and 1.0 μL template. Furthermore, specific amplification was verified using melting curve analysis, which always results in a single peak. See Fan and Xing (2016) for further details. The value of amplification efficiency was 97.03%, and the value of *R*^2^ was 0.997.

### 16S rRNA Gene High Throughput Sequencing and Data Processing

The DNA amplification used the bacterial primers 515f (5′-GTGCCAGCMGCCGCGG-3′) with barcode and 907r (5′-CCGTCAATTCMTTTRAGTTT-3′) (Lane, 1991). The library preparation and DNA sequencing were carried out using Illumina MiSeq with 2 × 250 bp protocol (Illumina, Inc., San Diego, CA, United States). The submission of raw sequence data was performed to ENA database (PRJEB22633), with accession numbers from SAMEA104433817 to SAMEA104433851.

The quality control of raw sequence data was carried out using Trimmomatic software (Bolger et al., 2014). The screening chimeras of clean sequences and operational taxonomic unit (OTU) clustering at 97% similarity were performed using Usearch (Edgar et al., 2011). Using the RDP Classifier, the taxonomic data were assigned to each representative sequence against the SILVA database (release_128) at 80% similarity (Quast et al., 2013; Cole et al., 2014). The above analyses were performed on QIIME platform (Caporaso et al., 2010).

### Data Analysis

The P flux through the interface of sediment-water was estimated according to Fick’s diffusion law using (Equation 1; Boudreau, 1996).

(1) $$F=-\frac{\varphi \cdot D_{0}}{\theta^{2}}\frac{\partial C}{\partial_{z}}$$

where

 *F* is the P flux (mg SRP m^−2^ day^−1^),

 φ is the sediment porosity (dimensionless),

 *D*_0_ is the solute diffusion coefficient for dihydrogen phosphate (cm^2^ s^−1^),

 θ is the sediment tortuosity (dimensionless),

 *C* is the solute concentration (mg L^−1^) of phosphate,

 *z* is the depth (m).

 Tortuosity was calculated using (Equation 2).

(2) $$\theta^{2}=1-\ln(\varphi^{2})$$

The SRP concentration gradient at the sediment-water surface, $\frac{\partial C}{\partial_{z}}$ |_z = 0_, is represented by the slope of line fitted to the top-most linear part of the relationship between concentration and depth.

Kolmogorov–Smirnov test was used to test the normality of data (Lilliefors, 1967). If the data distribution was normal, the significance of correlation between the parameters was evaluated using Pearson method (Hauke and Kossowski, 2011). Otherwise, the Spearman method was used. The differences among the sampling sites on each sampling season were determined using one-way analysis of variance (ANOVA) with Duncan’s *post hoc* tests (Day and Quinn, 1989). The analyses were carried out using SPSS version 16.0 with significant level of 0.05 (SPSS Inc., Chicago).

The normalization of OTU data was performed with 28,932 as the number for each sample sequences. The diversity of bacterial community was investigated as described in a previous work, and included indices of Shannon index, Simpson index, Chao index, diversity coverage, and observed OTUs number (Fan and Xing, 2016; Supplementary Table S2).

Significance in the difference of bacterial community structure among the three sampling zones was tested based upon the analysis of similarities (ANOSIM) at OTU level. The multivariate constrained ordination method was used to analyze the relationship between bacterial species and environmental parameters. The redundancy analysis (RDA) was selected, as the largest axis length was 2.62, which was determined using detrended correspondence analysis. Monte Carlo permutations was used to test the significance of environmental factors (permu = 999). The above analyses were performed using vegan package (Dixon, 2003) on the platform of R for statistical computing (Ihaka and Gentleman, 1996).

The taxa potentially driving the P flux difference between summer and other seasons was identified using the method of linear discriminant analysis effect size (LEfSe) in the three sampling sites (Segata et al., 2011). On the website^1^, the LEfSe was conducted at the genus level, and the significance level was set to be 0.05.

## Results

### Basic Physicochemical Properties of Sediment

Concentrations of sediment TP, TN, TOC, and LOI differed significantly among the three sampling zones, and were found in the following descending order: the macrophyte-dominated bay > the cyanobacteria-dominated bay > the cyanobacteria-dominated open water zone, except for TP and LOI in April (Table 1). The average concentrations of TP, TN, TOC, and LOI in the macrophyte-dominated bay sediment were 0.63 g kg^−1^, 2.98 g kg^−1^, 105.75 g kg^−1^, and 11.05%, respectively, while the corresponding concentrations in the cyanobacteria-dominated bay sediment were 0.53 g kg^−1^, 0.90 g kg^−1^, 52.29 g kg^−1^, and 4.99%, respectively. In the sediment of the cyanobacteria-dominated open water lake zone, the average concentrations in TP, TN, TOC, and LOI were 0.46 g kg^−1^, 0.69 g kg^−1^, 39.76 g kg^−1^, and 4.22%, respectively.

**Table 1:** Summary of the main characteristics of Lake Taihu sediments (a, b, and c indicate results of Duncan’s *post hoc* tests significant at 0.05 level).

Sample		TP (g kg^−1^)	TN (g kg^−1^)	TOC (g kg^−1^)	LOI (%)
April	H	0.54 ± 0.11 a	0.65 ± 0.21 b	42.22 ± 4.68 c	5.17 ± 0.73 b
	M	0.52 ± 0.01 a	0.82 ± 0.09 b	54.86 ± 3.55 b	5.02 ± 0.66 b
	D	0.58 ± 0.00 a	2.58 ± 0.15 a	102.01 ± 0.48 a	10.49 ± 1.69 a
July	H	0.47 ± 0.05 b	0.67 ± 0.06 b	38.91 ± 2.14 b	4.43 ± 0.46 b
	M	0.52 ± 0.03 b	0.93 ± 0.23 b	49.64 ± 3.46 b	4.91 ± 0.20 b
	D	0.63 ± 0.02 a	3.21 ± 0.58 a	93.91 ± 19.03 a	12.07 ± 0.69 a
October	H	0.45 ± 0.02 c	0.79 ± 0.17 b	42.07 ± 4.85 c	4.13 ± 0.32 c
	M	0.55 ± 0.01 b	1.01 ± 0.01 b	55.18 ± 4.47 b	5.28 ± 0.56 b
	D	0.74 ± 0.03 a	2.71 ± 0.54 a	118.72 ± 5.17 a	11.44 ± 0.68 b
January	H	0.39 ± 0.01 b	0.64 ± 0.14 b	35.83 ± 2.80 c	3.16 ± 0.09 c
	M	0.55 ± 0.01 a	0.88 ± 0.03 b	48.60 ± 5.26 b	4.71 ± 0.74 b
	D	0.56 ± 0.03 a	3.43 ± 0.30 a	108.38 ± 3.12 a	10.20 ± 0.41 a
Total	H	0.46 ± 0.08 c	0.69 ± 0.15 b	39.76 ± 4.25 c	4.22 ± 0.85 c
	M	0.53 ± 0.02 b	0.90 ± 0.13 b	52.29 ± 4.82 b	4.99 ± 0.56 b
	D	0.63 ± 0.07 a	2.98 ± 0.52 a	105.75 ± 12.74 a	11.05 ± 1.15 a

*H, Huxin. M, Meiliangwan. D, Dongtaihu. all data presented are based on −20∼−30 mm sediment depth.*

**FIGURE 2:**
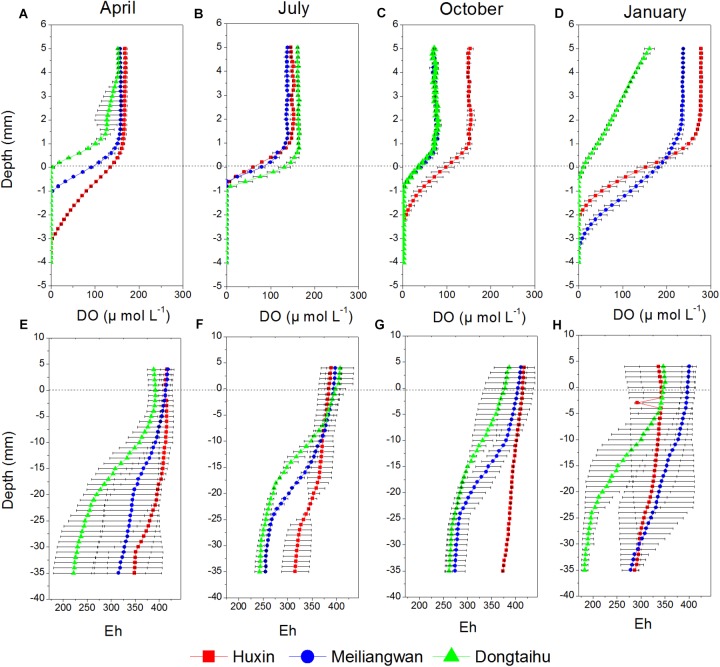
Vertical distribution of DO and Eh of sediment cores sampled from Huxin, Meiliangwan and Dongtaihu on April 2016 **(A,E)**, July 2016 **(B,F)**, October 2016 **(C,G)**, January 2017 **(D,H)**.

Dissolved oxygen (DO) permeating depths in the macrophyte-dominated bay sediment (−0.1 to −1.1 mm) were all lower than those in the cyanobacteria-dominated zone (−0.5 to −3.7 mm) for all the seasons, except for the summer when the DO permeating depths between the two contrasting zones changed little (Figure 2). The DO permeating depths tended to be higher in the sediment of the cyanobacteria-dominated open water lake zone, as compared to that of the cyanobacteria-dominated lake bay except in January (Figures 2A–D). The values of redox potential (Eh) in the macrophyte-dominated bay sediment (177.66–427.19) were lower than those of the cyanobacteria-dominated zone (253.93–431.17) (Figures 2E–H). The Eh values in the sediment of the cyanobacteria-dominated lake bay were lower than those of the cyanobacteria-dominated open water zone except for the January.

### Distribution of SRP and Dissolved Fe

**FIGURE 3:**
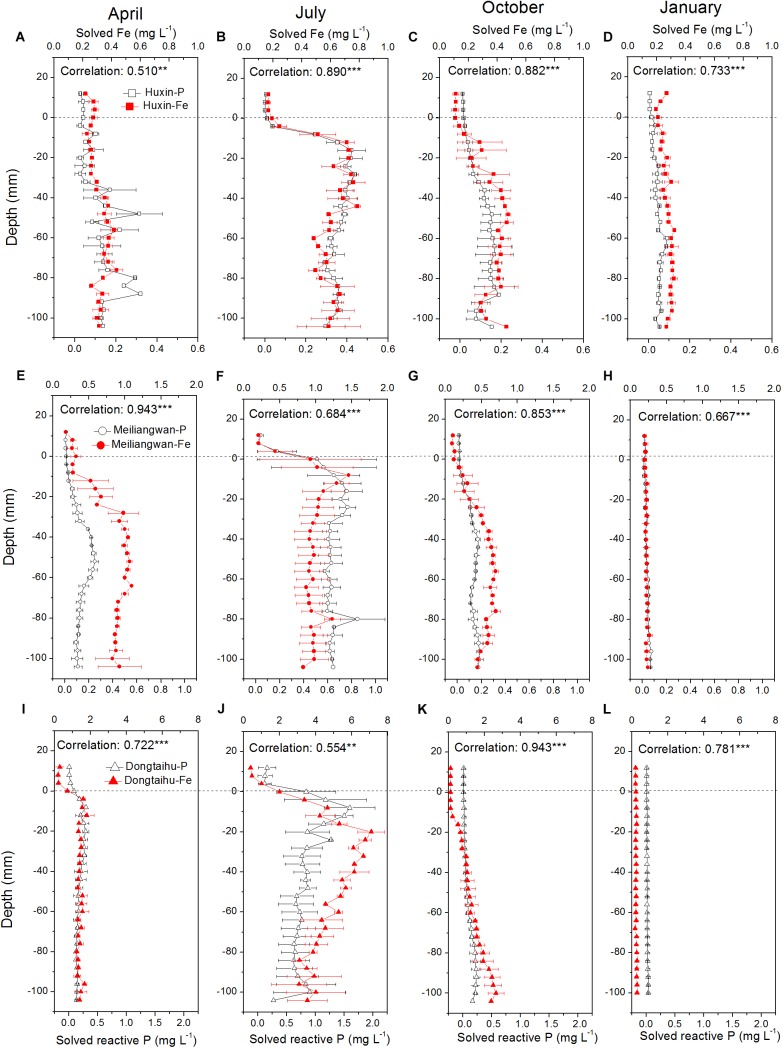
Vertical distribution of dissolved and reactive P, and dissolved Fe in the sediment cores with 4 mm interval sampled from Huxin, Meiliangwan and Dongtaihu on April 2016 **(A,E,I)**, July 2016 **(B,F,J)**, October 2016 **(C,G,K)**, January 2017 **(D,H,L)**. Correlation indicates tests between dissolved reactive P and dissolved Fe. Significant correlations between the two parameters are indicated with ^∗∗^ for 0.01 level and ^∗∗∗^ for 0.001 level.

The concentrations of dissolved Fe and SRP among the three sampling zone sediments followed the trend of being highest in the macrophyte-dominated bay, followed by the cyanobacteria-dominated bay, and lastly by the cyanobacteria-dominated open water zone (Figure 3). The vertical distributions of dissolved Fe and SRP in the three sampling sites changed with the season, with the highest concentrations in July (dissolved Fe = 7.82 mg L^−1^, SRP = 2.03 mg L^−1^) and the lowest in January (dissolved Fe = 0.26 mg L^−1^, SRP = 0.03 mg L^−1^). Meanwhile, the highest concentrations of dissolved Fe and SRP shifted to higher layer at the 0 to −30 mm depth of sediment in July, while in all other seasons, the highest values were found below −30 mm depth. The correlation between dissolved Fe and SRP was significant in the three sampling sites for each season with vertical resolution of 4.0 mm all with *p*-values lower than 0.001 except Huxin in April (*p* = 0.004) and Dongtaihu in July (*p* = 0.001).

Based on the above dissolved Fe and SRP data, the Fe/P ratio was further analyzed (Table 2). The molar ratios were significantly higher in the macrophyte-dominated bay (average values = 9.74) than those in the cyanobacteria-dominated bay (average values = 4.01) and open water zone (average values = 3.48). The P flux among the three sampling zone sediments was significantly different in April and July, while it changed marginally in October and January. The P fluxes were the highest in the sediment of the macrophyte-dominated bay, followed by the cyanobacteria-dominated bay and then the open water zone in April and July. The highest value of P flux (7.01 mg m^−2^ d^−1^) was detected in July.

**Table 2:** Molar Fe/P ratios and P flux of the three sampling sites in each month (a, b, and c indicate results of Duncan’s *post hoc* tests significant at 0.05 level).

Sample		Molar Fe/P ratios	P flux (mg m^−2^ day^−1^)
April	H	2.72 ± 0.85 b	0.04 ± 0.03 c
	M	6.13 ± 0.05 a	0.40 ± 0.13 b
	D	4.85 ± 1.16 ab	0.66 ± 0.16 a
July	H	1.54 ± 0.17 b	0.73 ± 0.06 b
	M	1.48 ± 0.20 b	2.22 ± 1.18 ab
	D	3.26 ± 0.49 a	4.33 ± 2.52 a
October	H	2.64 ± 0.80 b	0.20 ± 0.10 a
	M	2.15 ± 0.37 b	0.14 ± 0.02 a
	D	14.27 ± 0.74 a	0.12 ± 0.04 a
January	H	4.72 ± 2.14 ab	0.01 ± 0.01 a
	M	5.82 ± 3.85 b	0.01 ± 0.00 a
	D	12.47 ± 2.99 a	0.00 ± 0.00 a
Total	H	3.48 ± 2.30 b	0.24 ± 0.31 a
	M	4.01 ± 2.26 b	0.81 ± 1.12 a
	D	9.74 ± 4.50 a	1.45 ± 2.32 a

*H, Huxin; M, Meiliangwan; D, Dongtaihu.*

### Bacterial Abundance and Community Composition

**FIGURE 4:**
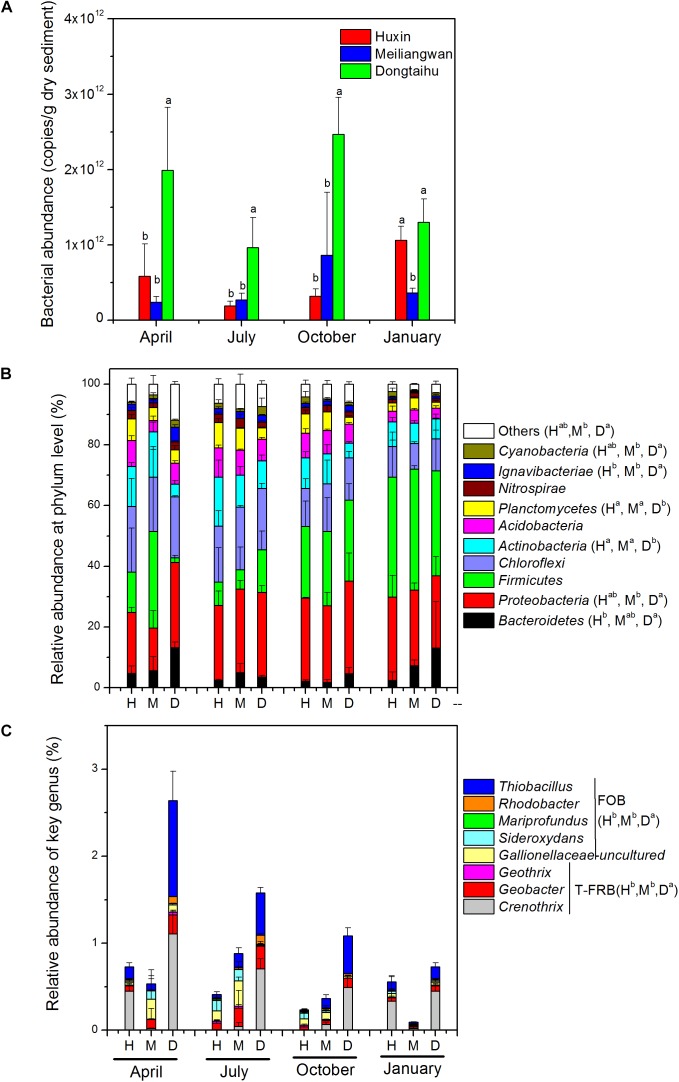
**(A)** Total bacterial abundance. **(B)** bacterial community composition at phylum level. **(C)** Genus-level classification of typical Fe(III)-reducing bacteria (T-FRB) and Fe(II)-oxidizing bacteria (FOB). H, Huxin; M, Meiliangwan; D, Dongtaihu; a, b, and c indicate results of Duncan’s *post hoc* tests at 0.05 level.

The results of qPCR indicated that the total bacterial abundance in the sediment of the macrophyte-dominated lake bay was significantly higher than those of the cyanobacteria-dominated bay or the open water zone (Figure 4A). Based upon the global ANOSIM comparison at the OTU level, the statistically significant difference (R: 0.4912, *p* = 0.001) was observed among the structures of overall bacterial communities of the three sampling sites. Phylum level classification revealed ten most dominant bacterial phyla (>1% of the total sample sequences), including *Proteobacteria* (25.05%), *Firmicutes* (22.38%), *Chloroflexi* (15.73%), *Actinobacteria* (9.48%), *Acidobacteria* (6.42%), *Bacteroidetes* (5.51%), *Planctomycetes* (4.82%), *Nitrospirae* (2.00%), *Ignavibacteriae*, (1.83%) and *Cyanobacteria* (1.42%; Figure 4B). *Proteobacteria*, *Bacteroidetes*, *Ignavibacteriae*, and *Cyanobacteria* were significantly more abundant in the macrophyte-dominated bay sediment than those in the cyanobacteria-dominated bay or the open water sediments, whereas *Actinobacteria* and *Planctomycetes* showed the opposite trend. Furthermore, the presences of typical Fe (III)-reducing bacteria, such as *Crenothrix*, *Geobacter*, and *Geothrix*, and some of the Fe (II)-oxidizing bacteria, including *Thiobacillus*, *Sideroxydans*, *Mariprofundus*, *Rhodobacter*, and uncultured *Gallionellaceae* (Figure 4C) were also detected. Both the typical Fe (III)-reducing and Fe (II)-oxidizing bacterial taxa were significantly higher in the macrophyte-dominated bay than those in the cyanobacteria-dominated bay and the open water sediments.

### Relationship Between the Bacteria and Physicochemical Factors

The abundances of total bacteria and typical Fe (II)/Fe (III) redox bacteria were significantly positively correlated with the nutrient factors (TOC, TN, TP, and LOI) but had a significant and negative correlation with those related to sediment redox state (DO permeating depth and Eh; Table 3). Furthermore, typical Fe(III)-reducing bacterial abundance was positively correlated with dissolved Fe (*p* = 0.040), while total bacterial abundance was positively correlated with the molar ratios of Fe/P (*p* < 0.001).

**Table 3:** Correlation analysis between abundance of bacteria (BA), typical Fe(III)-reducing bacteria (T-FRB), Fe(II)-oxidizing bacteria (FOB) and environmental factors of Lake Taihu sediment (^∗^, ^∗∗^, and ^∗∗∗^ indicate that the correlation is significant at 0.05, 0.01, and 0.001 level, respectively, and in bold).

Factors	BA	T-FRB	FOB
Dissolved Fe	0.310	**0.349^∗^**	0.094
Dissolved reactive P	0.012	0.205	0.213
Molar Fe/P ratio	**0.536^∗∗^**	0.253	0.141
P flux	−0.271	−0.025	0.080
DO	**−0.352^∗^**	**−0.489^∗∗^**	**−0.433^∗∗^**
Eh	**−0.554^∗∗^**	**−0.559^∗∗∗^**	**−0.433^∗∗^**
TOC	**0.627^∗∗∗^**	**0.619^∗∗∗^**	**0.550^∗∗^**
TN	**0.445^∗∗^**	**0.515^∗∗^**	**0.510^∗∗^**
TP	**0.660^∗∗∗^**	**0.662^∗∗∗^**	**0.581^∗∗∗^**
LOI	**0.516^∗∗^**	**0.643^∗∗∗^**	**0.692^∗∗∗^**

*All factors used the values of -20 ∼ -30 mm sediment depth except for P flux. DO, DO permeating depth in the sediment.*

**FIGURE 5:**
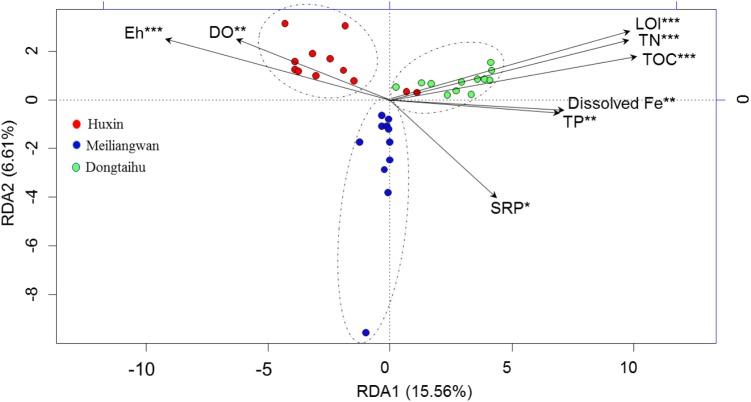
Results of redundancy analysis of the bacterial community and environmental factors based on −20 ∼ −30 mm sediment for all samples. SRP, dissolved reactive P. DO, DO permeating depth in the sediment. ^∗^Significant at 0.05 level. ^∗∗^Significant at 0.01 level. ^∗∗∗^Significant at 0.001 level.

The first axis of RDA explained 15.56% of the variance in bacterial community at the OTU level, while the second axis of RDA explained 6.61% based on the total samples (Figure 5). The Monte Carlo permutations for all the samples revealed that the distribution of bacterial community was significantly correlated with the SRP (*p* = 0.035), dissolved Fe (*p* = 0.009), and other environmental factors (TOC, TN, TP, LOI, Eh, and DO permeating depth).

### Summer Bacterial Representatives and Correlation With P Flux

**FIGURE 6:**
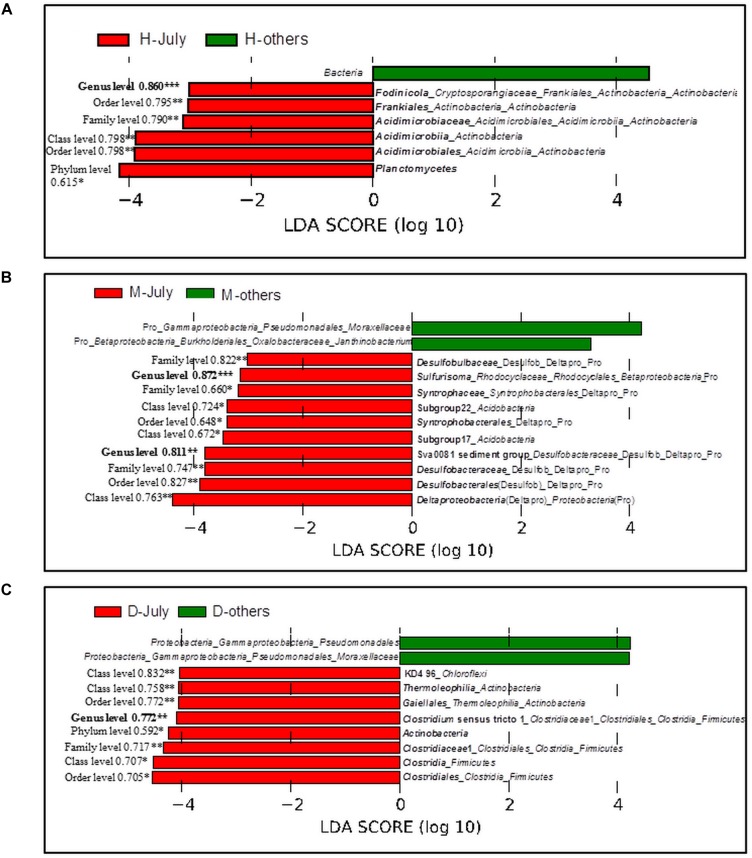
Linear Discriminant Analysis (LDA) using LefSe software with factorial Kruskal–Wallis test at 0.05 significance level. Bar plot shows the dominant bacterial groups in the sediments **(A)** Huxin, **(B)** Meiliangwan, **(C)**. Dongtaihu for July and the other 3 months (April, October, and January). H, Huxin; M, Meiliangwan; D, Dongtaihu; Numbers on the left of red bars were the result of correlation analysis between P flux and the relative abundance of corresponding taxa using all 4 month data, and significant correlation were indicated with ^∗^ for 0.05 level, ^∗∗^for 0.01 level, and ^∗∗∗^for 0.001 level.

The bacterial taxa, whose relative abundance was significantly higher in July than those in other three sampling months, were identified. In the sediment under the cyanobacteria-dominated open water, *Fodinicola*, *Acidimicrobiaceae*, and *Planctomycetes* were the representative bacterial groups of summer (Figure 6A). In the cyanobacteria-dominated bay sediment, Sva0081 sediment group, *Desulfobulbaceae*, *Sulfurisoma*, *Syntrophaceae*, and Subgroup17 and 22 of *Acidobacteria* were the representative bacterial groups in summer (Figure 6B). In the macrophyte-dominated bay sediment, *Clostridium* sensu stricto1, *Gaiellales*, and KD4_96 were the representative bacterial groups in summer (Figure 6C).

The relative abundances of representative bacterial groups in summer in the three sampling sites were all significantly correlated with the sediment P flux in all 4 months (Figure 6).

## Discussion

### Heterogeneity of SRP and Dissolved Fe in Sediments

In this study, the concentrations of SRP and dissolved Fe were the highest in the macrophyte-dominated bay sediment, followed by those in the cyanobacteria-dominated bay sediment, and finally, in the cyanobacteria-dominated open water sediment (Figure 3). The values of DO and Eh are the key environmental factors controlling the oxidation-reduction of Fe, affecting both Fe (II) availability and P mobility (Gu et al., 2016). Anaerobic conditions often lead to higher concentration of SRP and dissolved Fe in the sediments. In this study, the DO and Eh values were lower in the macrophyte-dominated bay sediment than those in the cyanobacteria-dominated lake zones (Figure 2). The historical external P loading in the cyanobacteria-dominated open water zone was lower than that in the cyanobacteria-dominated bay, which might be the main reason for lower concentrations of SRP and dissolved Fe in the sediment (Ding et al., 2015).

In addition, the molar ratio of dissolved Fe/P in the sediment of the macrophyte-dominated lake bay was significantly higher than that of the cyanobacteria-dominated lake zones (Table 2). The extent of P precipitation in waters can be governed by the stoichiometric Fe/P ratio during the shift from anoxic to oxic conditions and therefore, can be used as an indicator of the ability for P retention of lakes. In the oxidative hydrolysis of Fe and the concomitant precipitation of P, the precipitation of one P molecule needs a minimum of two Fe atoms (Blomqvist et al., 2004; Thibault et al., 2009). In this study, the Fe/P ratio was higher than 2 in all the four seasons in the macrophyte-dominated sediment, while it was lower than 2 in the cyanobacteria-dominated sediment in summer (Table 2). This could be one of the key reasons why the TP in aerobic or microaerobic overlying water was lower in the macrophyte-dominated zone than that in the cyanobacteria-dominated zone (Supplementary Table S1), although the SRP showed the opposite trend in the anaerobic sediment (Figure 3).

### Heterogeneity of Bacterial Community in the Sediments

The total bacterial abundance in the sediment of the macrophyte-dominated lake bay was significantly higher than those in the cyanobacteria-dominated lake bay or the open water zone sediments (Figure 4A). A significant difference in the composition of bacterial community was also observed among the cyanobacteria-dominated bay, the cyanobacteria-dominated open water zone sediment, and the macrophyte-dominated bay sediment (*p* = 0.001, Figure 5). In particular, *Proteobacteria*, *Bacteroidetes*, *Ignavibacteriae*, and *Cyanobacteria* were significantly higher in the macrophyte-dominated bay sediment, while *Actinobacteria* and *Planctomycetes* were the dominant taxa in the cyanobacteria-dominated zone (Figure 4B). The quantity and quality of natural organic matter are the most important factors impacting the abundance and composition of microbial community in lake sediments (Torremorell et al., 2015). The composition of organic matter in the cyanobacteria zone was very different from that in the aquatic macrophyte zone, and the accumulation of organic matter in the sediment could be higher in the macrophyte-dominated area because of the higher biomass of macrophyte than cyanobacteria (Zhang et al., 2013). This is consistent with TP, TN, TOC, and LOI being higher in the sediment of the macrophyte-dominated lake zone than those in the cyanobacteria-dominated lake zone (Table 1). Besides, the macrophytes can also affect the abundance and composition of bacterial community in the sediments, especially in the rhizosphere, through the excretion of root exudates, organic acids, and O_2_ (Lamers et al., 2012). Some previous studies have reported the heterogeneity of microbial community in Lake Taihu sediments, including both *Bacteria* and *Archaea* (Chen et al., 2015; Fan and Xing, 2016), methanogens (Fan and Wu, 2014), and ammonia- oxidizing prokaryotes (Wu et al., 2010).

In this study, the relative abundances of typical Fe (III)-reducing and Fe (II)-oxidizing bacteria in the sediment of the macrophyte-dominated lake zone were significantly higher than those in the cyanobacteria-dominated lake zone (Figure 4C). The higher decomposition of organic matter in the macrophyte-dominated sediment often resulted in anaerobic conditions and lower Eh value (Table 1 and Figure 2), which are suitable for the growth of typical Fe (III)-reducing bacteria (Konhauser et al., 2011). The O_2_ excretion meanwhile from macrophyte roots could build a micro-aerobic rhizosphere environment, which could benefit some Fe (II)-oxidizing bacteria only growing at the lowest possible O_2_ concentration, normally below 10 μM (Druschel et al., 2008).

### Potential Influences of Bacterial Community on the P and Fe Cycling

A broad diversity of microorganisms could catalyze iron reduction in freshwater environments (Lovley, 2006). It has been reported that Fe reduction contributes 44–67% of the anaerobic carbon decomposition in freshwater sediments (Hyun et al., 2009). In addition, some bacterial taxa could catalyze Fe oxidation in micro-aerobic and anaerobic environments (Emerson et al., 2010). Organic matter, DO and Eh are all important factors affecting Fe(III)-reducing and Fe(II)-oxidizing bacteria (Konhauser et al., 2011). Redox transformations of Fe could regulate the P mobility, and sediment P is released when solid-phase Fe(III) is transformed to dissolved Fe(II) (Jilbert and Slomp, 2013). However, to the best of our knowledge, very few studies have investigated how bacterial abundance and community composition is coupled to the cycling of Fe and P, especially in the field. In this study, the abundance of typical Fe(III)-reducing bacteria was significantly correlated with dissolved Fe (Table 3), and the bacterial diversity was significantly correlated with dissolved Fe and SRP (Figure 5). The bacterial decomposition of organic matter could have depleted the DO and lowered the Eh values (Table 3 and Figure 5), thus accelerating the Fe(III) reduction by the typical Fe(III)-reducing bacteria and releasing more SRP.

Several studies have suggested that much of the SRP released to the overlying water came from the sediment in summer, which promoted the cyanobacterial blooms in the cyanobacteria-dominated habitat (Xie et al., 2003; Gao et al., 2014). However, the mechanism behind this phenomenon remains unclear. In this study, the potential bacterial groups contributing to the high SRP release in summer were investigated (Table 2 and Figure 6). The relative abundances of *Acidimicrobiaceae*, *Fodinicola*, *and Planctomycetes* were significantly higher in summer, and they significantly correlated with the sediment P flux in the cyanobacteria-dominated open water sediment (Figure 6A). The members of *Acidimicrobiaceae* could oxidize ferrous iron or reduce the ferric iron, and were frequently detected in the environment polluted by metals/metalloids (Stackebrandt, 2014). Cultivable *Fodinicola*, such as *Fodinicola feengrottensis* (Carlsohn et al., 2008), was isolated from acidic and metal-containing rocks, indicating that they might participate in iron cycling. *Planctomycetes* harbors members of the anaerobic ammonium oxidation bacteria (Gu et al., 2017), and the significant correlation of *Planctomycetes* with the sediment P flux indicated the coupling cycles of Fe and N. Therefore, the bacterial group capable of Fe metabolism, such as *Acidimicrobiaceae*, was the potential bacterial group responsible for high SRP release in summer in the cyanobacteria-dominated open water zone sediment.

**FIGURE 7:**
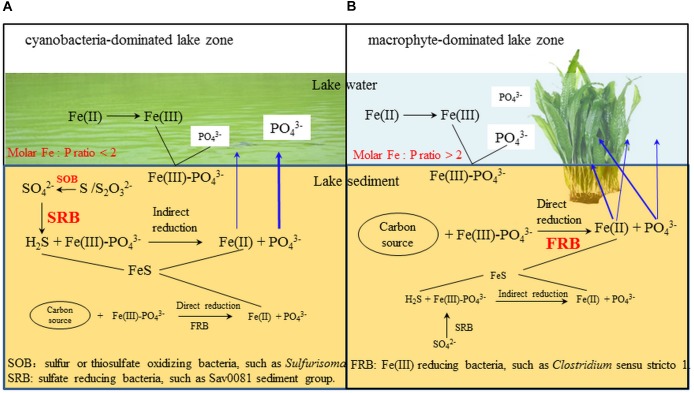
The potential microbial mechanism of P and Fe release from cyanobacteria- and macrophyte-dominated sediment in summer. Blue arrows indicate the fate of Fe and P. **(A)** cyanobacteria-dominated lake zone. **(B)** macrophyte-dominated lake zone.

In the cyanobacteria-dominated bay sediment, Sva0081 sediment group, *Desulfobulbaceae, Sulfurisoma, Syntrophaceae*, and Subgroup17 and 22 of *Acidobacteria* were the representative bacterial groups in summer, and their relative abundances were all significantly correlated with the sediment P flux (Figure 6B). The members of Sva0081 sediment group and *Desulfobulbaceae* could catalyze sulfate reduction (Timmers et al., 2016; Bao et al., 2018), while members of *Sulfurisoma* could oxidize thiosulfate and elemental sulfur (Kojima and Fukui, 2014). Furthermore, the uncultured Sav0081 sediment group could catalyze sulfate reduction associated with anaerobic oxidation of methane (Timmers et al., 2016), and the members of *Syntrophaceae* could be good partners of methanogens. Furthermore, the subgroups 17 and 22 of Acidobacteria were uncultured bacteria, and their roles in the sediment P flux need to be investigated. Previous studies have indicated that the bacterial lineages (*Desulfovibrio, Thiobacillus* and *Sulfuricurvum*) and *archaeal* (*Sulfolobales* and *Desulfurococcales*) capable of sulfur metabolism were dominant in the cyanobacterial blooms happening lake sediments (Chen et al., 2015; Fan and Xing, 2016). Sulfate reduction was considered as one of the key reasons for the high SRP release from the sediments (Roden and Edmonds, 1997). Because sulfide simultaneously reduces Fe (III) and precipitates Fe as FeS, it reduces the availability of Fe to bind P in both the ferric and ferrous solids (Rozan et al., 2002). Abundant labile organic matter, provided by the settling of dead cyanobacteria, DO depletion due to decomposition, and lower Eh could induce activity of sulfate reducing bacteria, such as Sva0081 sediment group and *Desulfobulbaceae*, contributing to high SRP release in summer in the cyanobacteria-dominated bay (Figure 7A).

In the macrophyte-dominated bay sediment, *Clostridium* sensu stricto1, *Gaiellales*, and KD4_96 affiliated to *Chloroflexi* were identified as the potential bacterial groups responsible for the higher sediment P flux in summer (Figure 6C). *Clostridium* sensu stricto 1 was considered as the true representative of the genus *Clostridium*, and the members of *Clostridium* sensu stricto 1 could degrade natural organic matters and produce low-molecular weight organic acids under strictly anaerobic environment (Gupta and Gao, 2009). Furthermore, the members of *Gaiellales* could utilize several organic compounds (Albuquerque and da Costa, 2014). KD4_96 affiliated to *Chloroflexi* was uncultured bacteria, and their roles in the sediment P flux need to be further investigated. In the macrophyte-dominated zone, the plant dead biomass and partially decomposed detritus settle on the surface of sediment, which leads to the formation of organic slime (Rejmankova and Houdkova, 2006). Therefore, the sediment is the main medium for the degradation and transformation of organic matter. Most of the anaerobic bacteria could couple oxidation of organic carbon with the reduction of Fe (III) (Konhauser et al., 2011). In summer, higher labile root exudates could activate the anaerobic organic matter-degrading bacteria, such as *Clostridium* sensu stricto 1, which might directly participate in the reduction of Fe (III) or indirectly provide substrates (such as, readily degradable organic acids) for other Fe (III)-reducing bacteria (Lovley, 2006; Gupta and Gao, 2009), which might in turn result in an increase in SRP release in the macrophyte-dominated zone (Figure 7B).

## Conclusion

The results showed higher SRP, dissolved Fe and Fe/P ratio in the macrophyte-dominated sediments than those in the cyanobacteria-dominated sediments. Consistent with this were the significantly higher abundances of total and typical Fe (II)/Fe (III) redox bacteria in the macrophyte-dominated sediments than those in the cyanobacteria-dominated sediments. Moreover, the work revealed potential influences of bacterial abundance and diversity on Fe and P cycles in the sediment, with dissolved Fe, SRP and Fe/P ratio particularly being significantly affected. In addition, the potential bacterial groups contributing to the higher P flux in summer were also identified in this work. In the cyanobacteria-dominated open water zone, the bacteria capable of Fe metabolism, such as *Acidimicrobiaceae*, were responsible for high SRP release in summer. In the cyanobacteria-dominated bay, sulfate reducing bacteria, such as Sva0081 sediment group and *Desulfobulbaceae* potentially contributed to higher P flux. In the macrophyte-dominated zones, anaerobic organic matter-degrading bacteria, *Clostridium* sensu stricto 1, were potentially the responsible bacterial group. This study enhances the understanding of effects of bacterial community on Fe and P coupling cycles in sediments under different primary producer-dominated ecosystems.

## Author Contributions

XF was responsible for the microbial experiment, data processing and writing. SD was responsible for the designing the experiment and guiding the writing. DT assisted with the revision of the manuscript. All the other authors were responsible for the experiment except the microbiological investigation.

## Conflict of Interest Statement

The authors declare that the research was conducted in the absence of any commercial or financial relationships that could be construed as a potential conflict of interest.

## Footnotes

1 http://huttenhower.sph.harvard.edu/galaxy

## Supplementary Material

TABLE S1 Seasonal variation of overlying water physicochemical properties at the three sampling sites (H, Huxin; M, Meiliangwan; D, Dongtaihu).

TABLE S2 α Diversity of bacterial communities of Lake Taihu sediment at the three sampling sites (H, Huxin; M, Meiliangwan; D, Dongtaihu).
