# Supplementary material for: Different Influences of Bacterial Communities on Fe (III) Reduction and Phosphorus Availability in Sediments of the Cyanobacteria- and Macrophyte-Dominated Zones
Source: Front Microbiol. 2018 Nov 14;9:2636. doi: 10.3389/fmicb.2018.02636 (PMC6247781; doi:10.3389/fmicb.2018.02636)
Supplement: TABLE S1 — Seasonal variation of overlying water physicochemical properties at the three sampling sites (H, Huxin; M, Meiliangwan; D, Dongtaihu). [file Data_Sheet_1.pdf]

Supplementary Material

Different Influences of bacterial communities on Fe(III) reduction and phosphorus availability in sediments of the cyanobacteria- and macrophyte-dominated zones

Xianfang Fan[1], Shiming Ding[1]*, Mengdan Gong [1,2], Musong Chen[1], ShuaiShuai Gao[1,2], Zengfeng Jin[1,2], Daniel C.W. Tsange[3]

*** Correspondence:** Shiming Ding: smding@niglas.ac.cn

Supplementary Tables

Two tables were put on Supplementary Material.

Table S1. Seasonal variation of overlying water physicochemical properties at the three sampling sites (H: Huxin; M: Meiliangwan; D: Dongtaihu).

Sample		T (℃)	DO (mg L-1)	pH	TP (mg L^{-1})	TN (mg L^{-1})	Chl-a (mg m^{-3})
April	H	19.1	8.64	8.33	0.057	2.36	6.7
	M	21.6	12.16	8.93	0.051	1.81	20.2
	D	19.3	9.20	8.42	0.041	1.60	18.2
July	H	29.9	4.08	8.44	0.030	1.38	15.0
	M	29.5	12.64	8.53	0.147	2.66	127.3
	D	29.1	6.47	8.35	0.113	1.46	51.7
October	H	19.1	8.02	8.23	0.123	2.38	22.4
	M	19.4	6.85	7.94	0.098	1.82	18.1
	D	17.8	8.31	8.25	0.059	1.38	10.2
January	H	8.1	11.19	8.47	0.109	1.41	14.1
	M	8.4	10.88	8.45	0.071	0.10	13.4
	D	7.9	8.50	8.16	0.056	1.55	5.3
Total	H	19.05±8.90	7.98±2.94	8.37±0.11	0.080±0.044	1.88±0.56	14.55±6.42
	M	19.73±8.71	10.63±2.63	8.46±0.41	0.091±0.042	1.60±1.07	44.75±55.11
	D	18.53±8.68	8.12±1.16	8.30±0.11	0.067±0.032	1.50±0.10	21.35±20.92

Table S2. α Diversity of bacterial communities of Lake Taihu sediment at the three sampling sites (H: Huxin; M: Meiliangwan; D: Dongtaihu).

Sample		OTU	Chao	Shannon	Simpson	Coverage
April	H	2805±296	3830±301	6.31±0.74	0.02±0.03	0.98±0.00
	M	2148±626	3231±581	5.08±1.48	0.10±0.11	0.98±0.01
	D	2749±68	3697±117	6.70±0.03	0.00±0.00	0.98±0.00
July	H	2832±302	4043±207	6.52±0.29	0.01±0.00	0.98±0.00
	M	2782±269	3645±654	6.62±0.32	0.01±0.00	0.99±0.00
	D	2196±62	3241±17	6.03±0.15	0.01±0.00	0.98±0.00
October	H	2494±307	3661±482	5.68±0.60	0.03±0.03	0.98±0.00
	M	2210±352	3162±380	5.56±0.84	0.04±0.05	0.97±0.01
	D	2040±161	3059±94	5.12±0.40	0.06±0.02	0.98±0.01
January	H	2006±234	3577±346	4.27±0.62	0.13±0.08	0.98±0.00
	M	1560±69	2579±164	4.22±0.15	0.12±0.01	0.99±0.00
	D	2046±316	3439±605	4.28±0.47	0.13±0.06	0.98±0.00
Total	H	2534±425	3778±351	5.70±1.05	0.05±0.06	0.98±0.00
	M	2120±544	3110±540	5.26±1.15	0.07±0.07	0.98±0.01
	D	2258±341	3359±363	5.54±0.99	0.05±0.06	0.98±0.00
